# Intracochlear Fluid Pressure Changes Related to the Insertional Speed of a CI Electrode

**DOI:** 10.1155/2014/507241

**Published:** 2014-07-16

**Authors:** I. Todt, P. Mittmann, A. Ernst

**Affiliations:** Department of Otolaryngology at UKB, Unfallkrankenhaus Berlin, Warenerstaße, 12683 Berlin, Germany

## Abstract

*Introduction*. To preserve residual hearing the atraumaticity of the cochlea electrode insertion has become a focus of cochlear implant research. In addition to other factors, the speed of insertion is thought to be a contributing factor in the concept of atraumatic implantation. The aim of our study was to observe intracochlear fluid pressure changes due to different insertional speeds of an implant electrode in a cochlear model. *Materials and Methods*. The experiments were performed using an artificial cochlear model. A linear actuator was mounted on an Advanced Bionics IJ insertional tool. The intracochlear fluid pressure was recorded through a pressure sensor which was placed in the helicotrema area. Defined insertions were randomly performed with speeds of 0.1 mm/sec, 0.25 mm/sec, 0.5 mm/sec, 1 mm/sec, and 2 mm/sec. *Results*. A direct correlation between speed and pressure was observed. Mean maximum values of intracochlear fluid pressure varied between 0.41 mm Hg and 1.27 mm Hg. *Conclusion*. We provide the first results of fluid pressure changes due to insertional speeds of CI electrodes in a cochlear model. A relationship between the insertional speed and intracochlear fluid pressure was observed. Further experiments are needed to apply these results to the in vivo situation.

## 1. Introduction

The indication for cochlear implantation has changed over the years from complete deafness with bilateral implantation to patients with substantial residual hearing. This development was possible following trials in new patient groups, as well as by the development of new electrode designs and refining of the surgical technique. To achieve the aim of hearing preservation, different studies with variable concepts and results have been published [1–3].

The intracochlear force applied by the insertion of the cochlear implant electrode is a point of interest since the required amount of force to achieve disruption of the basilar membrane was estimated for the first time [4]. Since that time, a number of studies have estimated the insertional force of different electrodes and different speeds of insertion [5–8]. Unfortunately, contradictory results were found when comparing the insertional force and the clinical degree of hearing preservation with different types of electrodes. While electrodes with a low insertional force (0.008 N) [6] using specific insertional techniques (Cochlear Advance Contour, AOS technique) showed limited hearing preservational properties [3], electrodes with a higher insertional force (0.032 N, [9], Flex EAS) showed higher rates of hearing preservation [2]. Besides the mechanical properties of the electrodes, other forces could explain those findings.

Clinically, the speed of insertion has been described as a factor that contributes to the rate of hearing preservation [10]. Roland [8] made measurements of intracochlear fluid pressure changes (ICFP) during the insertion of a CI electrode and discussed this as a possible cause for lesions within the microstructures of the inner ear.

The aim of the present study was therefore to estimate the effects of the insertional speed of electrode insertion on ICFP in a cochlear model.

## 2. Material and Methods

### 2.1. Electrode and Linear Actuator

For all experiments, Advanced Bionics IJ electrodes were used. IJ electrodes were inserted through a metal tube with the regular insertion tool. The electromagnetic linear actuator (IP4, Berlin, Germany) was mounted on the regular insertional tool and fixed in front of the cochlear model to perform hand-free insertion.

Tested insertional speeds were 0.1 mm/sec, 0.25 mm/sec, 0.5 mm/sec, 1 mm/sec, and 2 mm/sec. All different speeds were tested three times in a random variation of the different speeds.

### 2.2. Pressure Sensor

The intracochlear pressure was measured using a microoptical pressure sensor developed by Olson [11]. Details about the design, fabrication, and capacity can be found in the literature [11]. Basically, the tip of the pressure sensor is a hollow glass tube sealed on one end by a plastic thin film diaphragm coated with a reflective surface of evaporated gold. An optical fiber is located in the glass tube at a small distance (50–100 *μ*m) to from diaphragm tip. The optical fiber is attached to an LED light source and to a photodiode sensor. Light from the LED source reaches the sensor tip of the optical fiber, fans out as it exits the fiber, and is reflected by the gold-covered flexible diaphragm. The reflected light is sensed by the photodiode. Small pressure-induced distance displacements of the diaphragm modulate the intensity of reflected light. The sensor is connected to a module that is linked to a computer. Evolution software was used to record the intracochlear pressure. The temporal resolution of the sensor was 300 measurements per second.

### 2.3. Preparation of the Cochlear Model

**Figure 1:**
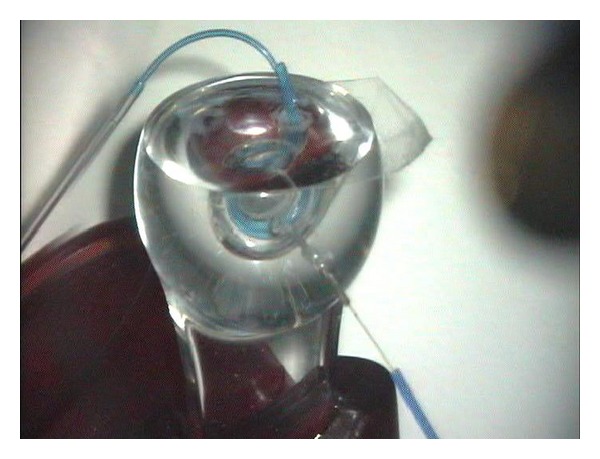
Cochlear model with positioned sensor and an inserted probe electrode.

These experiments were performed using a synthetic, transparent, artificial cochlear model (Figure 1). The opening of the cochlear model (cochleostomy) had a diameter of 1.5 mm. In the helicotrema area of the cochlear model, an extra channel was drilled to be slightly larger (about 200 *μ*m) than the sensor tip to insert the pressure sensor. After the pressure sensor was inserted, the cochlea was filled with water and the position of the sensor within the channel was fixed and sealed with fibrin glue. The sensor was placed within the channel in such a way that the tip was not in contact with the edge of the channel or the ground. Afterwards, the cochlea was microscopically controlled to exclude any enclosed air bubbles.

### 2.4. Measurements

The sensor was calibrated in the cochlea and the initial value was set to zero. A measurement was considered useful if the measured value after finalization of the experiment returned to the initial value. After every insertion, the model was refilled with water and checked microscopically for any enclosed air bubbles.

## 3. Results

**Figure 2:**
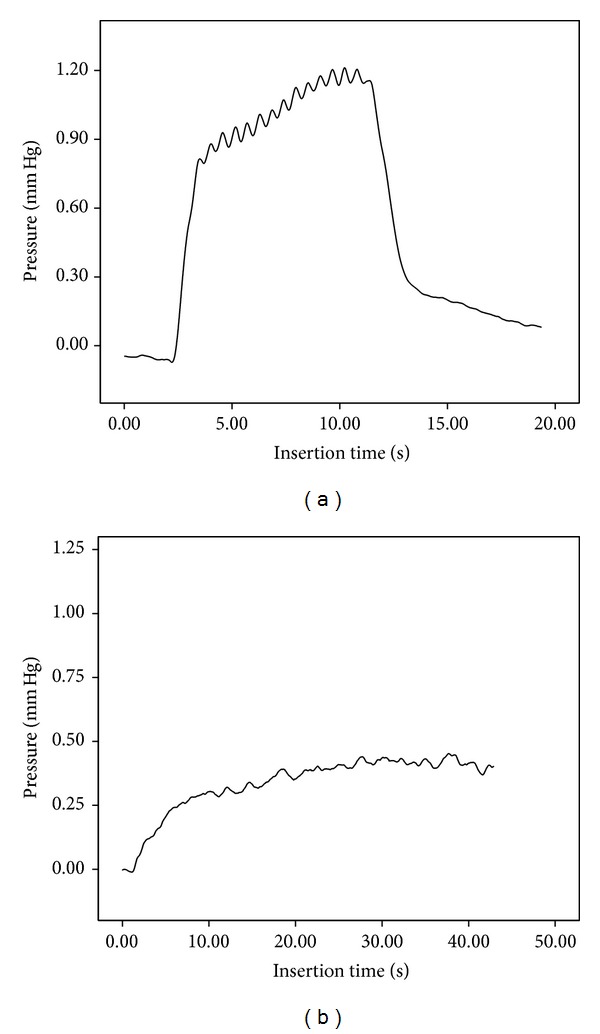
Example of the measurement of 2 mm/sec (a) and 0.25 mm/sec (b) insertions and recorded ICFP.

With an insertional speed of 0.1 mm/sec the pressure was 0.43 mm Hg, SD 0.058 mm Hg. For a speed of 0.25 mm/sec, we observed 0.51 mm Hg, SD 0.076 mm Hg. With a speed of 0.5 mm/sec, we measured 0.79 mm Hg, SD 0.156 mm Hg. For a speed of 1 mm/sec, the pressure was 1.2 mm Hg, SD 0.14 mm Hg. At 2 mm/sec, the pressure was 1.27 mm Hg, SD 0.110 mm Hg (Figure 2). The low values of the standard deviation indicate the good reproducibility of the experimental setup.

**Figure 3:**
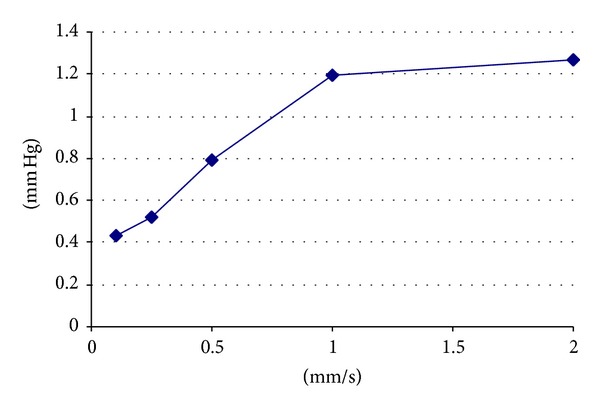
Mean maximum ICFP against different insertional speeds of cochlear implant electrodes.

A comparison of the different insertion speeds showed Gaussian curve-like behavior of the mean maximum ICFP measured (Figure 3). The observed sinusoidal curve is assumed to be related to the electromagnetic sinusoidal push behavior of the linear actuator.

## 4. Discussion

Different factors can be assumed to contribute to hearing preserving cochlear implant surgery. Variations in opening the round window have been described and have shown a significant impact on the transmission of intracochlear fluid pressure force in a cochlear implant model [12]. Various forms of application (i.v., middle ear, topical) and different medications (triamcinolone, dexamethasone, prednisolone) are used and thought to be important factors for the preservation of residual hearing [13, 14]. Occlusion of access to the cochlea to prevent the secondary outflow of lymph has been performed by specific cut fascia, artificial shields, or cork-like solutions [15].

The speed of insertion as a variable for the preservation of hearing was first discussed by Kontorinis et al. [5] and clinically shown by Rajan et al. [10] who observed speed-dependent variations in the degree of hearing preservation and vestibular function. These findings were successfully included in a hearing preservation concept for midmodiolar electrodes [16].

A number of studies evaluated the insertional force of different electrodes and different speeds of insertion [5–8]. Unfortunately, contradictory results were found when comparing the insertional force and the clinical degree of hearing preservation with different types of electrodes. While electrodes with a low insertional force (0.008 N) [6] using specific insertional techniques (Cochlear Advance Contour, AOS technique) showed limited hearing preservational properties [3], electrodes with higher insertional force (0.032 N, [9], Flex EAS) showed better rates of hearing preservation [2]. Beside mechanical properties, other forces could contribute to this observation. Roland discussed fluid pressure-related lesions for the first time.

The fluid pressure showed an increasing pattern from basal to apical or with an increasing depth of insertion (Figure 2). This angle flattened with decreasing speed (Figure 2). Based on this Gaussian pattern of fluid pressure in relation to the insertional speed, an insertion speed of 0.25 mm/sec could be recommended for the model. Todt and Ernst [16] used similar insertional speeds for the successful preservation of residual hearing with the Advanced Bionics High Focus MS electrode (Stäfa, Switzerland).

The transformation of this speed into in vivo ICFP might be difficult. The estimated ICFP values might be higher since the relationship between the used model and the volume of the electrode is related to a smaller intracochlear scalar volume in vivo modified. Additionally, the degree of round window opening might be a factor that influences the leakage of fluid while the electrodes pass through the cochlea; this could influence the ICFP toward higher values. The natural main pathway for pressure equilibration, the cochlear aqueduct, is highly variable in terms of size, patency, and fluid resistance [17, 18]. It was our intention to simulate this route by a relatively large model opening/cochleostomy (1.5 mm). Regarding the probability of intracochlear trauma related to hydrostatic pressure changes, slow fluid pressure changes are separate from fast sound pressure-related fluid changes. The literature related to this topic is limited and does not offer clear answers [19, 20].

Sound-induced intracochlear pressure changes are widely described in the literature. In a gerbil model, a maximum of 10 Pa was measured in the scala vestibuli with a stimulus of 90 dB SPL at 15 kHz in the outer ear canal [21]. In the scala tympani (3.5 mm from the stapes with 80 dB SPL at the stapes), the pressure varied up to 90 dB SPL (0.63 Pa) near the basilar membrane [22]. Physiological hydrostatic pressure has been described in the guinea pig at 200 Pa with variations between −110 and 700 Pa [23].

The observed ICFP values in our model range from 0.41 mm Hg to maximum values of 1.27 mm Hg (169 Pa or 0.024 psi). Further observations in animal models and human observations are needed to relate these results to possible intracochlear trauma.

We provide the first results of fluid pressure changes due to insertional speeds of CI electrodes in a cochlear model. A relationship between the insertional speed and ICFP changes could be observed. Further experiments are needed to apply our results to the in vivo positioning of a cochlear implant electrode in a human cochlea.
